# Versatile extracellular vesicle-mediated information transfer: intercellular synchronization of differentiation and of cellular phenotypes, and future perspectives

**DOI:** 10.1186/s41232-024-00318-5

**Published:** 2024-01-15

**Authors:** Tomohiro Minakawa, Jun K. Yamashita

**Affiliations:** Department of Cellular and Tissue Communication, Graduate School of Medicine, The University of Tokyo, Tokyo, 113-8655 Japan

**Keywords:** Extracellular vesicle, Intercellular communication, Phenotypic synchronization of cells, Embryonic stem cell

## Abstract

In recent years, extracellular vesicles (EVs) have attracted significant attention as carriers in intercellular communication. The vast array of information contained within EVs is critical for various cellular activities, such as proliferation and differentiation of multiple cell types. Moreover, EVs are being employed in disease diagnostics, implicated in disease etiology, and have shown promise in tissue repair. Recently, a phenomenon has been discovered in which cellular phenotypes, including the progression of differentiation, are synchronized among cells via EVs. This synchronization could be prevalent in widespread different situations in embryogenesis and tissue organization and maintenance. Given the increasing research on multi-cellular tissues and organoids, the role of EV-mediated intercellular communication has become increasingly crucial. This review begins with fundamental knowledge of EVs and then discusses recent findings, various modes of information transfer via EVs, and synchronization of cellular phenotypes.

## Subgroups and markers of EVs

**Fig. 1:**
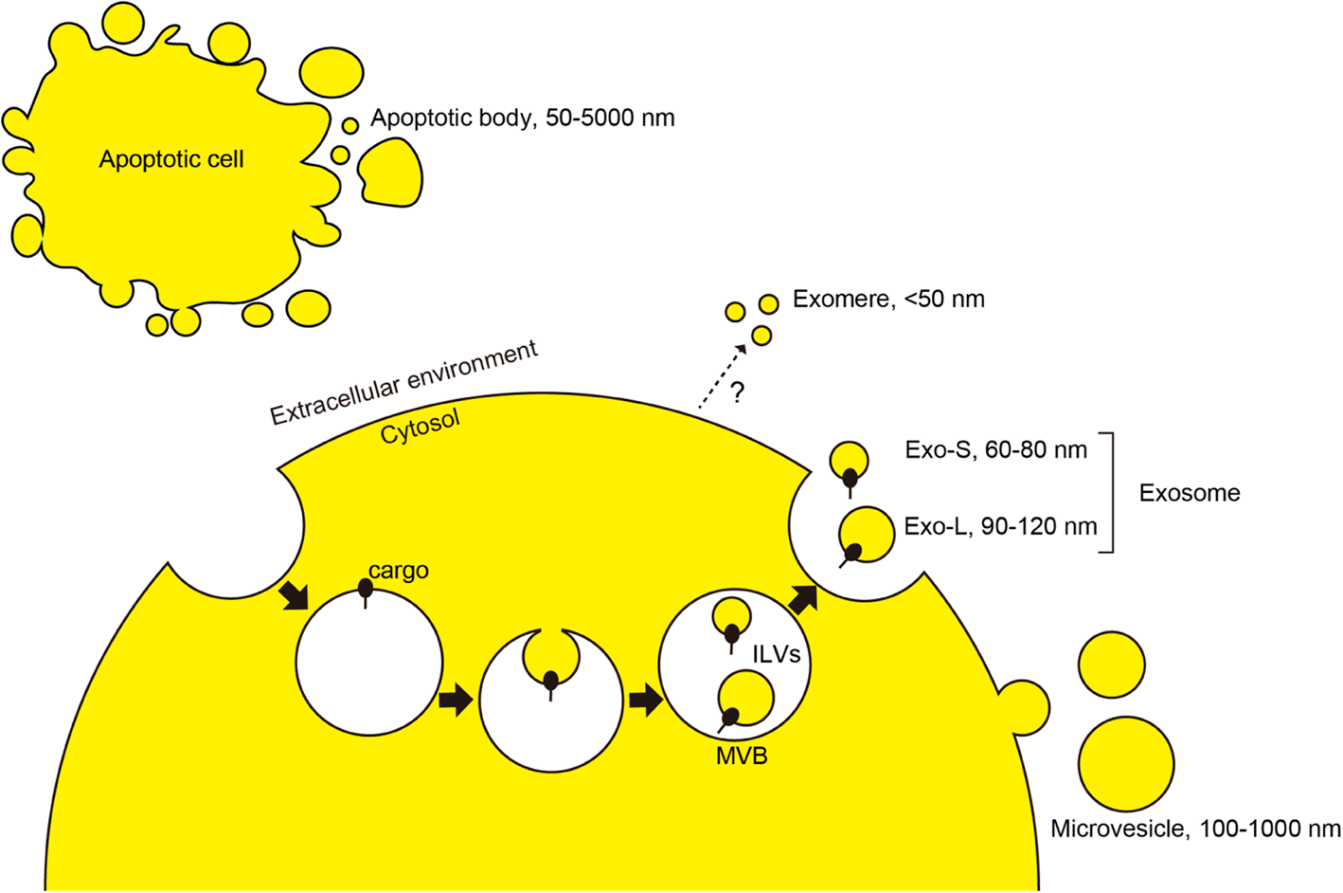
Classification of EVs

Extracellular vesicles (EVs) are membranous structures released by cells and categorized into several subgroups with their distinct formation mechanisms; exosomes are formed from the budding of endosomes, microvesicles are directly budded from the cell membrane [1], and apoptotic bodies are produced by the breakdown of apoptotic cells [2, 3] (Fig. 1). Zhang and colleagues further defined three distinct subpopulations of exosomes: small exosomes (Exo-S, 60–80 nm), large exosomes (Exo-L, 90–120 nm), and exomeres (< 50 nm). Unlike other vesicles, exomeres are not surrounded by a lipid bilayer and are not enriched with ESCRT-related molecules, making their generation mechanism elusive [4]. It is important to note that in this study, the Exo-L fraction contains a significant amount of Annexin A1, which has been reported as a characteristic marker of microvesicles [5]. This suggests the possibility that microvesicles may also be present in the Exo-L fraction. Therefore, the risk of relying solely on size for exosome fractionation should be considered.

**Fig. 2:**
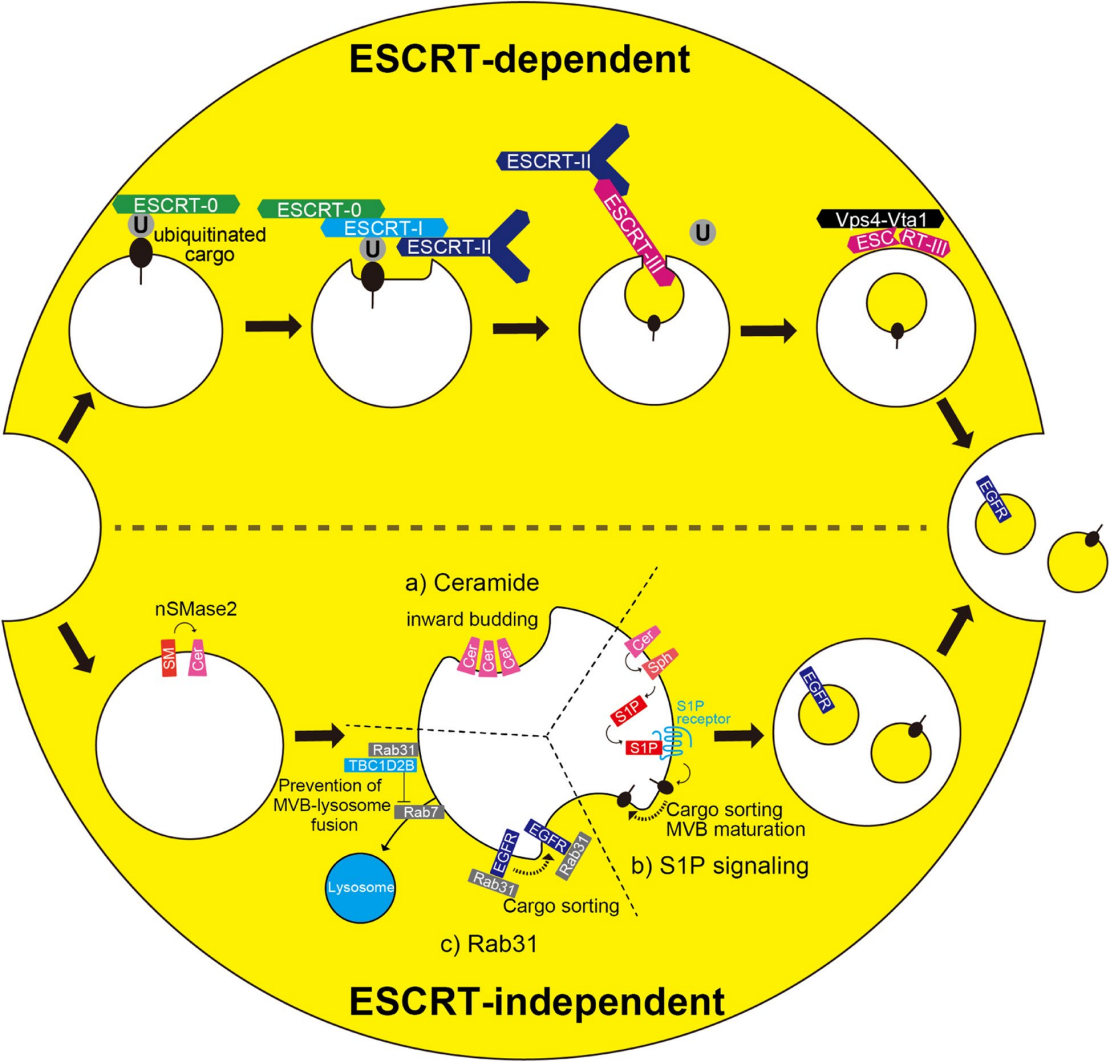
ESCRT-dependent and ESCRT-independent exosome biogenesis. ESCRT-dependent exosome biogenesis (upper side): ESCRT-0 recognizes ubiquitinated cargo and binds it to the endosomal membrane [12]. ESCRT-0 recruits ESCRT-I, which along with ESCRT-II stabilizes the neck of the forming vesicle. Subsequently, ESCRT-III narrows the neck [13]. At this stage, the cargo is deubiquitinated [14]. Upon recruitment of the Vps4-Vga1 complex, the scission of the vesicle neck by ESCRT-III begins, and ultimately ESCRT-III is disassembled [13]. For detailed information on the individual subunits of ESCRT, refer to Henne et al. [14] and Williams et al. [15]. ESCRT-independent exosome biogenesis (lower side): **a** nSMase2 catalyzes the conversion of sphingomyelin to ceramide. It is hypothesized that cone-shaped ceramide accumulates locally, leading to the formation of ceramide-enriched microdomains that induce membrane curvature [8, 10, 16]. **b** Ceramide is metabolized to sphingosine, which is subsequently converted to sphingosine-1-phosphate (S1P). S1P is involved in the sorting of cargo into ILVs. S1P activates the S1P receptor, which helps in the maturation of MVBs [9]. **c** Rab31 sorts epidermal growth factor receptor (EGFR) into ILVs [10]. Rab31 recruits TBC1D2B, reducing the activity of Rab7 and inhibiting the fusion of MVBs with lysosomes. As a result, the inhibition of Rab7 promotes exosome secretion [11]. SM, sphingomyelin; Cer, ceramide; Sph, sphingosine; S1P, sphingosine-1-phosphate

Exosomes are generated through both ESCRT (endosomal sorting complexes required for transport)-dependent and ESCRT-independent mechanisms (Fig. 2). In the ESCRT-dependent mechanisms, the ESCRT complexes catalyze the formation of multivesicular bodies (MVBs) by invagination of the endosomal limiting membrane [6]. Some ESCRT components are suggested to selectively act on subpopulations of MVBs or intraluminal vesicles (ILVs) destined to be secreted as exosomes [7]. ESCRT-independent exosome generation requires the production of ceramides by the neutral sphingomyelinase 2 (nSMase2), which hydrolyzes sphingomyelin into ceramides. These ceramides then trigger the budding of exosome vesicles into MVBs [8]. Furthermore, the metabolic product of ceramide, sphingosine-1-phosphate (S1P), is implicated in the cargo sorting and the maturation of MVBs [9]. Tetraspanins, Rab proteins, and flotillin-1 are shared between both ESCRT-dependent and independent pathways [10]. On the other hand, within the Rab family, Rab31 is involved in cargo sorting, supporting ESCRT-independent exosome biogenesis. Additionally, Rab31 promotes exosome secretion by inhibiting the fusion of MVBs with lysosomes through Rab7 inhibition [11].

**Fig. 3:**
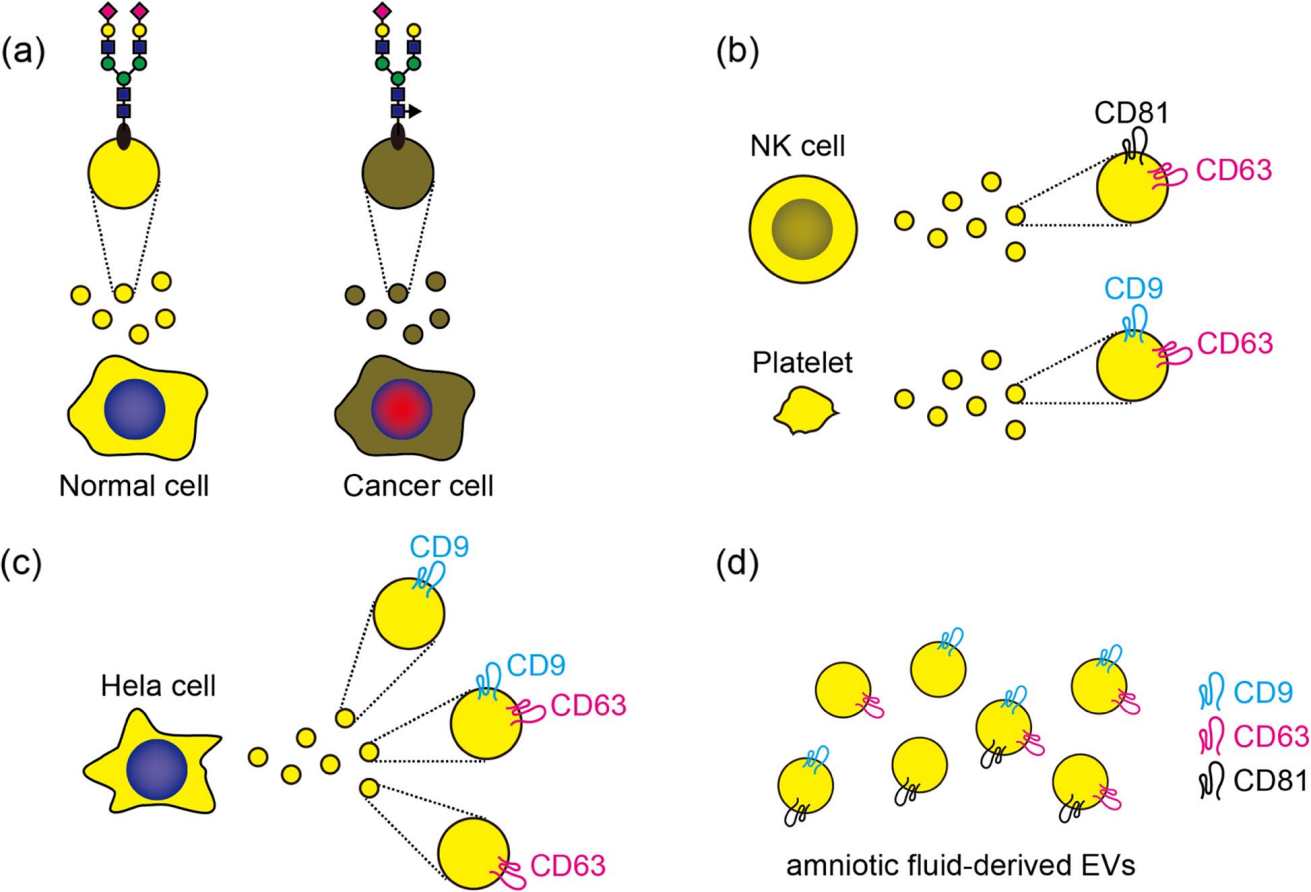
Glycosylation modification and tetraspanins on the surface of EVs. **a** Glycosylation modifications are altered in cancer cells. (Based on Shimoda et al. [18]). **b** Natural killer (NK) cell-derived EVs contain CD63 and CD81, but not CD9. On the other hand, platelet-derived EVs contain CD9 and CD63 but scarcely contain CD81 [20]. **c** There are HeLa cell-derived EVs that are CD9 single positive, CD63 single positive, and double positive [21]. **d** In amniotic fluid-derived EVs, some contain only one CD9, CD63, or CD81, while others contain multiple of them [22].

Once released from cells, distinguishing among the various subgroups of EVs, such as exosomes, microvesicles, and apoptotic bodies, becomes difficult. The International Society for Extracellular Vesicles (ISEV) has proposed categorizing them by size into small EVs (typically less than 100 nm or 200 nm), and medium/large EVs (greater than 200 nm), according to the Minimal Information for Studies of Extracellular Vesicles (MISEV2018) guidelines [17]. In this review, we generally use the term ‘EV’ unless specifically referring to a particular subgroup, especially exosomes. EV membranes are composed of various lipids, and various proteins and glycans are expressed on EV membranes. Many lipids and proteins are glycosylated. These glycosylation modifications are altered by cancer (Fig. 3a) [18, 19]. The expression profiles of such components vary depending on the cell types even in commonly used EV biomarkers like CD9, CD63, and CD81 (Fig. 3b) [20]. Furthermore, heterogeneity has been suggested even within EVs derived from the same cell type or source, namely the presence of EVs that are single positive, double positive, and triple positive for CD9, CD63, and CD81 has been reported (Fig. 3c, d) [21, 22]. Recently, it was found that syntenin-1 is the most common and consistently included in the proteome of exosomes derived from different cell lines, and was also identified in exosomes recovered from various species. It has been identified in exosomes from plasma, urine, breast milk, and saliva [23]. These results suggest that syntenin-1 could be used as a unique biomarker to distinguish exosomes purified from human biofluids from other EVs. Specific integrins expressed on exosomes recognize specific distant tissues/cells, and tumor-derived exosomes taken up by organ-specific cells prepare the pre-metastatic niche. Exosomes expressing integrins α6β4 and α6β1 bind to fibroblasts and epithelial cells in the lungs, governing tumor metastasis to the lung, while exosomes expressing integrin αvβ5 specifically bind to Kupffer cells, mediating liver metastasis [24].

## Contents of EVs

EVs were originally considered to serve the role of expelling unwanted cellular components. Indeed, it has been reported that they dispose of defective proteins, unnecessary proteins, and harmful DNA, thereby maintaining cellular homeostasis [25, 26]. With the recent realization that EVs play a role in intercellular communication [27], there has been an explosive increase in reports on their roles in various disease states including cancer, analyses of the cargo of EVs released from various cells, and their reparative effects on damaged tissues. EVs contain mRNA, microRNA (miRNA, miR), non-coding RNA (ncRNA), proteins, and lipids [28], with some reports suggesting the inclusion of mitochondria [29, 30]. While numerous studies report the presence of DNA in EVs, it has been noted that the DNA content within EVs is relatively low, and most detected DNA may be adhering to the surface of EVs or embedded in non-vesicular structures [5, 31]. EVs contain only a small amount of miRNA, and even the most abundant miRNA is detected at an average of only one copy per 121 EVs [32]. In another study, specific viral miRNA in EVs from virus-infected cells was found at a frequency of only one copy per 300 to 16,000 EVs [33]. Even EVs derived from the same cell are not constant in their contents but would be heterogeneous. A proteomic analysis of EVs has revealed the diversity of contents across various subpopulations of EVs [34]. The heterogeneity of EV contents should be further explored and discussed in future single EV analyses.

## EV uptake and information transmission mechanisms

**Fig. 4:**
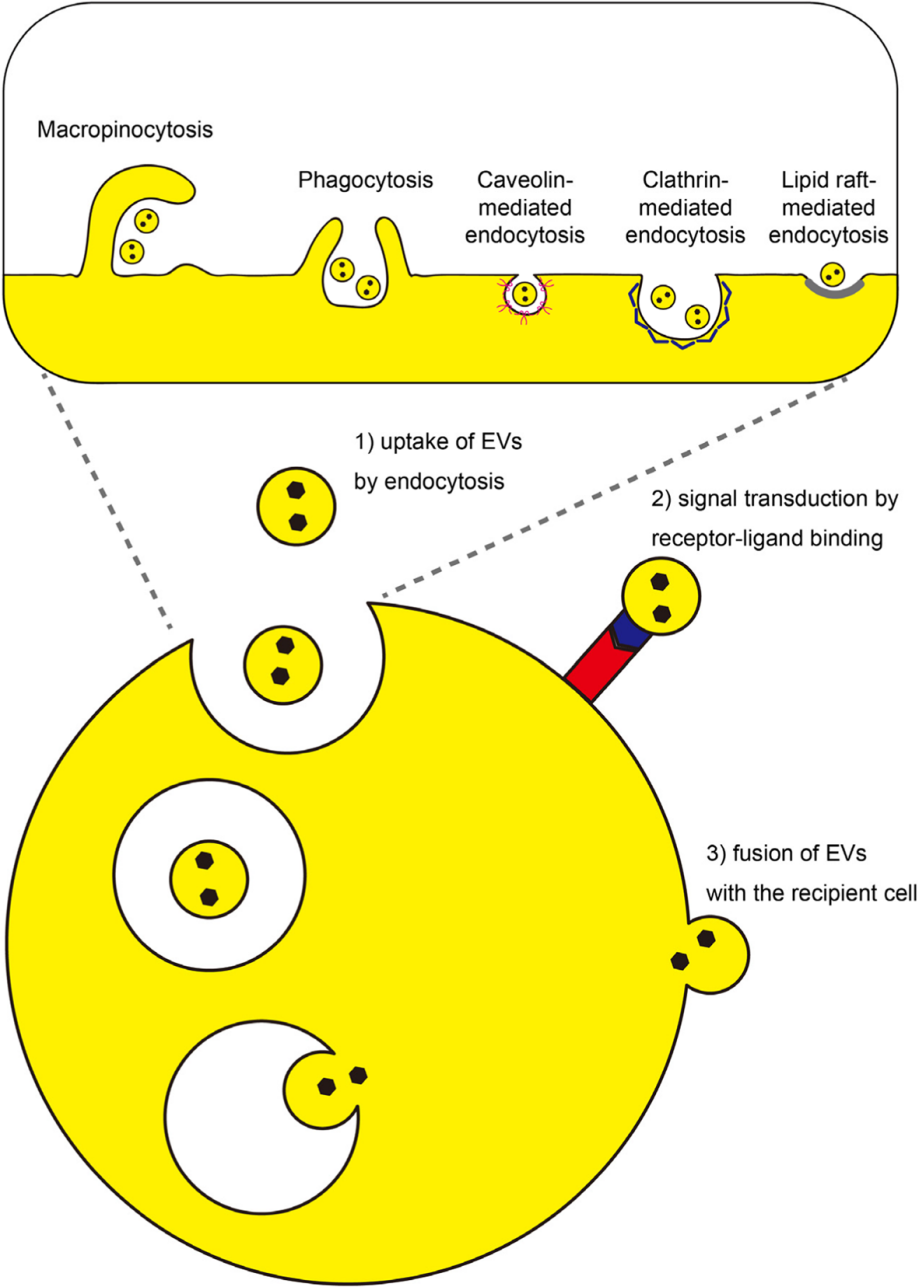
Three ways in which EVs send information to recipient cells. (Based in part on van Niel et al. [35])

EVs communicate with recipient cells through three primary mechanisms: (1) uptake of EVs by endocytosis, (2) signal transduction by receptor-ligand binding on the cell membrane, and (3) fusion of EVs with the recipient cell (Fig. 4). EVs that have reached the surface of the recipient cell membrane are taken up by clathrin-dependent endocytosis, caveolin-dependent endocytosis, lipid raft-dependent endocytosis, macropinocytosis, or phagocytosis [35]. After being taken up into endosomes, there is still little known about how the contents of EVs are released into the cytoplasm. A recent study by Joshi et al. demonstrated that the EV membrane fuses with the endosome/lysosome membrane under acidic conditions and releases its contents into the cytoplasm [36]. While this study did not observe EVs fusing with the cell membrane and releasing their contents into the cytoplasm, such a mechanism cannot be ruled out. As another example, Polanco et al. demonstrated a mechanism by which EVs containing tau protein, thought to be involved in Alzheimer’s disease, escape from endosomes. After EVs were taken up into endosomes, the degradation of endolysosomes increased the permeability of the endolysosomes, causing tau to leak into the cytoplasm and inducing tau aggregation [37]. Conversely, there are viewpoints challenging the efficiency of EVs in delivering cargo to the cytoplasm of recipient cells, suggesting that the cargo might not be functional or that the process is highly inefficient [33, 38]. Approximately 50,000 EVs per cell were incubated for 4 h, but the fusion of EVs with the plasma membrane or endosomal membrane of recipient cells was either extremely low or not detected [33]. In experiments using EVs incorporated with the virus-derived fusion protein VSV-G (vesicular stomatitis virus glycoprotein), which dramatically increases the efficiency of cargo transport to the cytoplasm, approximately 100,000 EVs per cell were incubated for 24 h, yet no function of miRNA was observed [33]. This inefficiency might be attributed to the low copy number of miRNA present in EVs. Proteins such as β-lactamase reporter and tetracycline transactivator, which were overexpressed, were clearly observed to function only when transported by EVs incorporated with VSV-G [33, 38]. These results suggest that a significantly larger amount of EVs or engineered EVs with improved membrane fusion capability may be required for effective cargo delivery. Additionally, instances of signal transduction independent of EV uptake have been documented. One of the earliest examples of signal transduction by receptor-ligand binding on the cell membrane involves EVs derived from B cells or dendritic cells that could present antigens to T cells and induce a specific antigenic response [39, 40]. It was discovered that angiopoietin-2 on the surface of EVs binds to the Tie2 receptor on recipient cells and activates the signal [41]. Laminin and fibronectin on EVs released from the inner cell mass (ICM) interact with integrins on the surface of the trophoblast, promoting trophoblast migration and embryo implantation [42].

## The multifaceted roles of EVs in molecular dynamics and signaling

There are several instances that the dynamics of molecules change when they are transported by EVs, compared to when soluble factors or ligands exist individually. Regarding the distribution of morphogens, the widely accepted model of gradient formation by passive diffusion cannot explain the specificity to certain target cells, the dynamics of long-range distribution, and the formation of intracellular and extracellular gradients [43]. It has been shown that Hedgehog (Hh) is secreted in an ESCRT-dependent manner within EVs moving along cytonemes (a type of filopodia) to create a gradient within Drosophila tissues [44, 45]. Additionally, it has been reported that Hh is transported long distances by EVs through cytonemes [44]. This mechanism produces a distribution of Hh different from that by passive diffusion. Some Wnts and Hhs undergo lipid modifications (palmitoylation or cholesterol modification) that are essential for signal transmission but can impair their free diffusion in the extracellular environment. Therefore, packaging in vesicles is required for the long-range action of lipid-modified morphogens [46, 47]. Notch is a transmembrane protein, and its intracellular domain is cleaved and downstream signaling is activated after binding to Delta on the surface of directly adjacent cells. Alternatively, a model has been proposed in which Delta on the surface of EVs triggers the activation of Notch signaling in the recipient cell [48, 49], suggesting the possibility of activating Notch signaling in distant cells without direct contact. Within the mouse embryo, rotating cilia create a fluid flow. By this fluid flow, EVs containing Sonic hedgehog and retinoic acid are transported to the left side of the embryo, influencing the determination of the left–right axis [50].

## Synchronization of cell differentiation through EVs

**Fig. 5:**
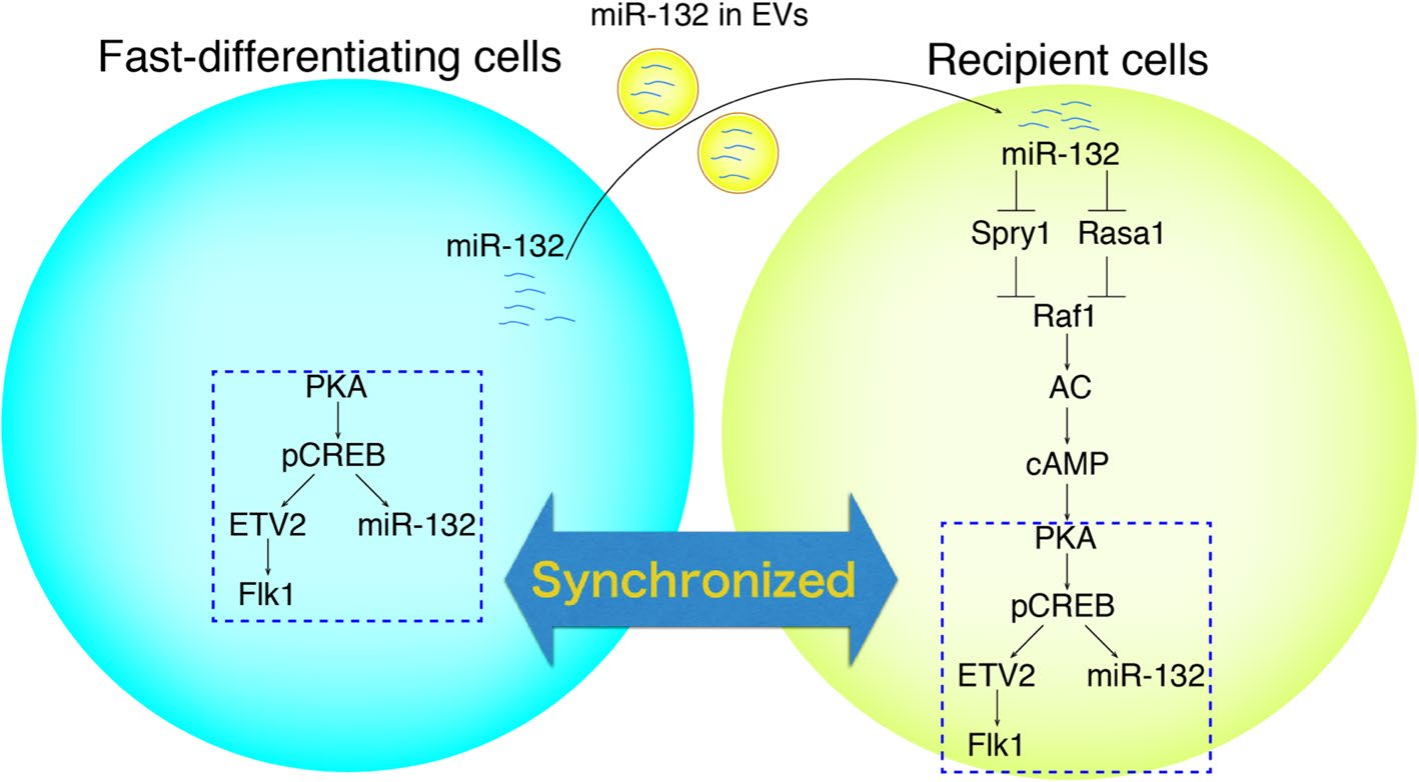
Schematic of the PSyC mechanism including EV secretion and miR-132 delivery. From fast-differentiating cells, miR-132 is transferred through EVs to surrounding recipient cells, where it inhibits Spry1 and Rasa1 to transmit the signal. As a result, the differentiation mechanism is synchronized in the fast-differentiating cells and recipient cells [53]

During development, cells coordinate their differentiation in a way that must align their fate determination and synchronize their differentiation stages with those of surrounding cells. While numerous soluble factors inducing differentiation have been reported, there are many instances where the cells producing these factors and the cells induced by them belong to distinct lineages. For example, during vasculogenesis in early development, vascular endothelial growth factor (VEGF) is a potent soluble factor that induces differentiation from mesoderm to vascular endothelial cells, yet its cell source is the endoderm [51]. Another example can be seen in chicken embryos, where bone morphogenetic protein (BMP) produced by the dorsal aorta prompts the differentiation of neural crest cells into adrenal medulla cells [52]. It has been challenging to explain the mechanism by which cells of the same lineage synchronize their differentiation with surrounding cells through soluble factors. Our recent research has unveiled a novel mechanism for how neighboring cells synchronize their phenotypes with each other and this synchronization is mediated through EVs [53]. Our discovery centers on the synchronization of cells in differentiation, particularly focusing on the coordination of fate determination towards mesoderm and the synchronization of the differentiation progression. In order to prove this, it was necessary to create an intentional gap in the degree of differentiation progress. For this purpose, we used a method we previously reported, where we intentionally accelerated the differentiation of embryonic stem cells (ESCs) into mesodermal cells by activating Protein Kinase A (PKA) [54]. In the established ESC line (PKA-ESCs), we can express activated PKA in a drug-controlled manner (Tet-OFF). When we culture Control-ESCs alone, which has the same differentiation speed as the wild type, less than 20% of the cells become Flk1 positive mesoderm cells only by day 4.5. On the other hand, when we culture PKA-ESCs alone and activate PKA under doxycycline-free (Dox-) conditions, more than 20% of Flk1 positive mesoderm cells in total cells appear from day 2.5 of differentiation. When we create a mixed aggregate of PKA-ESCs and Control-ESCs and co-culture them under differentiation conditions, the differentiation of Control-ESCs accelerates to catch up with PKA-ESCs, reaching a mesoderm positivity rate of 40% at day 3.5. We consider that this phenomenon can be defined as ‘phenotypic synchronization of cells (PSyC)’ (Fig. 5) [53]. When we added an EV inhibitor (an inhibitor of nSMase2 essential for exosome synthesis), Manumycin A or GW4869, to the mixed aggregate of PKA-ESCs and Control-ESCs, only the differentiation of Control-ESCs was inhibited. When we collected EVs from PKA-ESCs (PKA-ESC-EVs) and added them to Control-ESCs during a single culture, mesoderm differentiation was strongly promoted. When we added PKA-ESC-EV to mouse embryos and performed ex vivo culture, beating cardiomyocytes, a mesodermal derivative, was induced. To analyze the functional molecules contained in PKA-ESC-EV, we performed microRNA sequencing and found that miR-132 was particularly potent. We found that when artificial nanoparticles containing miR-132 were applied to cells, they induced differentiation into mesoderm. Moreover, when added to mouse embryos, they induced the differentiation of cardiomyocytes. These results demonstrate that it is possible to use the molecules inside EVs for cellular phenotypic synchronization. This synchronization was notably less efficient in a co-culture system using a transwell, which created a physical distance between PKA-ESCs and Control-ESCs. Also, when we labeled PKA-ESC-EVs with a fluorescence probe, we found that the efficiency of EV reaching Control-ESCs was markedly lower in the transwell system compared to mixed aggregate and 2D co-culture. This could be due to the fact that EVs should be immediately taken up by nearby cells as soon as they are released. From these observations, it was inferred that the delivery of EVs, especially the exchange of EVs between adjacent cells, is important for the phenotypic synchronization. Currently, we are exploring a new mode of cellular communication, focusing on the direct vesicle exchanges between adjoining cells, primarily using live imaging. Another interesting finding is that when we added PKA-ESC-EVs, Control-ESCs differentiated into mesoderm, but at this time, the PDGFRα positivity rate increased depending on the concentration of EVs, while the Flk1 positivity rate tended to decrease. This suggested that EVs have the potential to fine-tune the orientation towards the axial mesoderm within the mesoderm. EVs have been found to contain tens of thousands of entities, including RNAs, ncRNAs, proteins, and more. Considering the additional presence of lipids, DNA fragments, surface ligands, and glycans, we believe that EVs can share high-order information that cannot be achieved by single molecules.

Phenomena that imply the involvement of phenotypic synchronization in differentiation have been reported in various environments and cell types (Table 1). When EVs collected from differentiated NSPCs (neural stem progenitor cells) were added to proliferating NSPCs, differentiation was induced [55]. Mesenchymal stem cells (MSCs) received daily treatments for a week with EVs from the neural cell line PC12. After treatment with EVs from PC12 cells, the MSCs exhibited a neuron-like morphology, and the expression of genes and proteins of neuronal markers increased [56]. In ex vivo experiments, the addition of EVs derived from corneal epithelial cells increased the expression levels of corneal epithelial markers, while the addition of EVs derived from conjunctival epithelial cells increased the expression levels of conjunctival epithelial markers [57]. When EVs derived from hair papilla cells were added to adipose-derived stem cells, the cells became more likely to acquire hair papilla-like characteristics [58]. The addition of macrophage-derived EVs to naive monocytes induced differentiation into macrophages [59]. When EVs derived from ESCs were supplied to Müller cells, these cells changed to a de-differentiated precursor cell phenotype [60]. Cardiac-derived EVs have been identified to enhance the expression of specific cardiac-associated genes, namely GATA-binding protein 4 (GATA4), T-box transcription factor (Tbx5), NK-2 transcription factor related, locus 5 (Nkx2.5), and cardiac troponin T (cTnT), within human mesenchymal stem cells (hMSC) [61]. Utilizing EVs extracted from embryonic stem cells (ESCs) undergoing cardiac differentiation has facilitated the direct reprogramming of fibroblasts into induced cardiomyocyte-like cells, with success rates above 60% [62].

**Table 1:** Instances where cells receiving EVs acquire phenotypes similar to those of the donor cells

Donor cells	Recipient cells	Effects	References
PKA-ESCs (Fast-differentiating ESCs)	Control-ESCs (Slow-differentiating ESCs)	Promotion of differentiation and catching up to the same stage of differentiation as PKA-ESCs	[53]
		Potent commitment to the mesodermal lineage, similar to PKA-ESCs	
Differentiated NSPCs	NSPCs	Neural differentiation	[55]
Neural cells	MSCs	Induction of neuron-like morphology	[56]
Corneal epithelial cells	Conjunctival epithelial cells	Increase in marker levels of corneal epithelial cells	[57]
Conjunctival epithelial cells	Corneal epithelial cells	Increase in marker levels of conjunctival epithelial cells	
Hair papilla cells	Adipose-derived stem cells	Induction of hair papilla-like characteristics	[58]
Macrophages	Naive monocytes	Macrophage differentiation	[59]
ESCs	Muller cells	De-differentiation	[60]
Cardiomyocytes	MSCs	Induction of cardiac gene expressions	[61]
ESCs differentiating into cardiomyocytes	Fibroblasts	Supporting cardiac differentiation	[62]

## Synchronization and maintenance of cellular phenotypes via EVs

We believe that EVs should also contribute to the maintenance of cellular homeostasis, mainly intrinsic properties, within tissues, not just differentiation. Each cell constantly exchanges information with its surroundings. One example is the complementation of missing molecules. When endothelial cells with a knocked-out gene were cultured with adipocytes, it was observed that mRNA of the knocked-out gene was supplied from adipocytes to endothelial cells, compensating for the deficiency [63]. There have also been reports of cases, i.e. EVs from undifferentiated cells improve the quality of undifferentiated cells [64], in the co-culture of porcine parthenogenetic embryos and cloned (nuclear transfer) embryos, mRNA of pluripotency genes was delivered via EVs, improving the in vitro development of cloned embryos [65]. We hypothesize that there may be diseases that arise from the breakdown of mechanisms to maintain such homeostasis.

## Future perspectives

Numerous co-culture experiments of different cell types have been conducted so far [66–68], and while changes in cellular phenotypes have been observed, the molecular mechanisms are complicated and many remain unclear. Not only between different cell types but also between the same cell types, there should be a great deal of intercellular communication via EVs, including mechanisms like synchronization, even if they are not immediately apparent. We expect the molecular mechanisms contributed by EVs to become increasingly clear in the coming years. Furthermore, recent years have seen rapid progress in research on organoids, three-dimensional structures composed of multiple types of cells, and assembloids, that connect organoids from different brain regions [69]. We anticipate that such research will enable the modeling of complex intercellular interactions, further deepening our understanding of intercellular communication via EVs in vivo. The roles and importance of vesicle-mediated intercellular information transfer are expected to gain further validation in the near future.

## Data Availability

Not applicable.

## Abbreviations

BMP: Bone morphogenetic protein
Cer: Ceramide
cTnT: Cardiac troponin T
Dox: Doxycycline
EGFR: Epidermal growth factor receptor
ESC: Embryonic stem cell
ESCRT: Endosomal sorting complexes required for transport
EV: Extracellular vesicle
GATA4: GATA-binding protein 4
Hh: Hedgehog
ICM: Inner cell mass
ILV: Intraluminal vesicle
ISEV: International Society for Extracellular Vesicles
miRNA, miR: MicroRNA
MISEV: Minimal information for studies of extracellular vesicles
MSC: Mesenchymal stem cell
MVB: Multivesicular bodies
ncRNA: Non-coding RNA
Nkx2.5: NK-2 transcription factor related, locus 5
nSMase2: Neutral sphingomyelinase 2
NK cell: Natural killer cell
NSPC: Neural stem progenitor cell
PKA: Protein kinase A
PSyC: Phenotypic synchronization of cells
S1P: Sphingosine-1-phosphate
SM: Sphingomyelin
Sph: Sphingosine
Tbx5: T-box transcription factor
VEGF: Vascular endothelial growth factor
VSV-G: Vesicular stomatitis virus glycoprotein
